# Protein kinases mediate increment of the phosphorylation of cyclic AMP -responsive element binding protein in spinal cord of rats following capsaicin injection

**DOI:** 10.1186/1744-8069-1-26

**Published:** 2005-09-13

**Authors:** Jing Wu, Guangxiao Su, Long Ma, Xuan Zhang, Yongzhong Lei, Junfa Li, Qing Lin, Li Fang

**Affiliations:** 1 Division of Neurosurgery, Department of Surgery, The University of Texas Medical Branch, Galveston, TX 77555-0517; USA; 2 Department of Neurology, University of Texas Health Science Center, Houston, TX77030-1501, USA; 3 Department of Neuroscience and Cell Biology, The University of Texas Medical Branch, Galveston, TX 77555-1043, USA; 4 Institute for Biomedical Science of Pain, Department of Neurobiology, Capital University of Medical Sciences, Beijing 100054, China

**Keywords:** Central sensitization, transcription factors, protein kinase cascade, nociception

## Abstract

**Background:**

Strong noxious stimuli cause plastic changes in spinal nociceptive neurons. Intracellular signal transduction pathways from cellular membrane to nucleus, which may further regulate gene expression by critical transcription factors, convey peripheral stimulation. Cyclic AMP-responsive element binding protein (CREB) is a well-characterized stimulus-induced transcription factor whose activation requires phosphorylation of the Serine-133 residue. Phospho-CREB can further induce gene transcription and strengthen synaptic transmission by the activation of the protein kinase cascades. However, little is known about the mechanisms by which CREB phosphorylation is regulated by protein kinases during nociception. This study was designed to use Western blot analysis to investigate the role of mitogen-activated protein (MAP)/extracellular signal-regulated kinase (ERK) kinase (MEK 1/2), PKA and PKC in regulating the phosphorylation of CREB in the spinal cord of rats following intraplantar capsaicin injection.

**Results:**

We found that capsaicin injection significantly increased the phosphorylation level of CREB in the ipsilateral side of the spinal cord. Pharmacological manipulation of MEK 1/2, PKA and PKC with their inhibitors (U0126, H89 and NPC 15473, respectively) significantly blocked this increment of CREB phosphorylation. However, the expression of CREB itself showed no change in any group.

**Conclusion:**

These findings suggest that the activation of intracellular MAP kinase, PKA and PKC cascades may contribute to the regulation of phospho-CREB in central nociceptive neurons following peripheral painful stimuli.

## Background

Peripheral tissue injuries lead to persistent pain which can be caused, in part, by the central sensitization of spinal sensory neurons. It reflects an amplified responsiveness of nociceptive neurons that involves long-lasting changes in synaptic transmission in the central nervous system [1-3]. The changed central synaptic plasticity may be initiated by activity-dependent intracellular biochemical pathways following noxious stimulation of the peripheral tissue [1-5].

It has been extensively reported that extracellular signals conveyed and transduced through the plasma membrane to the intracellular nucleus trigger the expression of several immediate early genes, such as c-*fos*, c-*Jun*, Egr 1 and CREB [6-13]. The activation of these critical transcription factors further initiates a cascade of biological changes in neural functioning through changes in gene expression. Cyclic AMP (cAMP)-responsive element binding protein (CREB) is one of the well-characterized stimulus-induced transcription factors. Stimulus-induced influx of calcium activates the second messenger systems which are believed to be important for the long-term alteration in CREB-related gene expression [6,8,14-17]. Furthermore, this event involves the binding of CREB to a specific sequence present in the promoter of many cAMP responsive genes. The transduction pathways are comprised of cascades of intracellular signaling protein kinases, such as the second messenger system, [4,5,9,18,19]. These kinases play an important role in the subsequent activation or phosphorylation of the transcription factor, CREB. Phospho-CREB-induced gene transcription and strengthened synaptic transmission were widely reported in neuronal processing of various extracellular signals [8,13,14,17,20-22]. For example, it was reported that an increased CREB phosphorylation of the Serine-133 residue is responsible for the induction of long-term potentiation (LTP) in CA1 in the hippocampus. Additionally, the coordinated phosphorylation of CREB was initiated by the rapid phosphorylation of MAP kinase, as well as other activated protein kinases, such as calcium/calmodulin protein-dependent kinase II (CaM kinase II), protein kinase A and C [6,9,17,22-24]. More recently, central sensitization of spinal nociceptive neurons to peripheral stimulus was extensively reported and the mechanism of central sensitization was found in a spinal cord form of LTP [2]. Earlier studies showed that intradermal capsaicin injection in experimental animals induces central sensitization, which resembles LTP in many respects [1,2,4,25,26].

Central sensitization involves several crucial events generated by the activation of protein kinases, such as CaM kinase II, MAP kinase, protein kinase A (PKA), protein kinase C (PKC) and protein kinase G (PKG) [2,4,5,9,15,18,19,27]. These important cellular events trigger further transactivator selective target substrates, such as CREB, to enhance gene regulation [26,28]. We have found increased phosphorylation of spinal CREB protein following capsaicin injection in rats. Although the findings reported that phosphorylation events can be regulated by nitric oxide, as well as CaM kinase II [6,13,29], it remains to be determined whether MAP kinase, PKA and PKC participate in the phosphorylation of CREB in spinal cord central sensitization.

The current study was designed to examine the contribution of MAP kinase, PKA and PKC in the regulation of CREB protein phosphorylation in the ipsilateral side of the cord during central sensitization triggered by intraplantar capsaicin injection in rats.

## Results

The results from this study demonstrated that the CREB protein and its phosphorylated form, phospho-CREB, were detected by immunoblot analysis in each group of animals. The molecular weight of CREB and phospho-CREB were seen at the 45 KD band.

**Figure 1:**
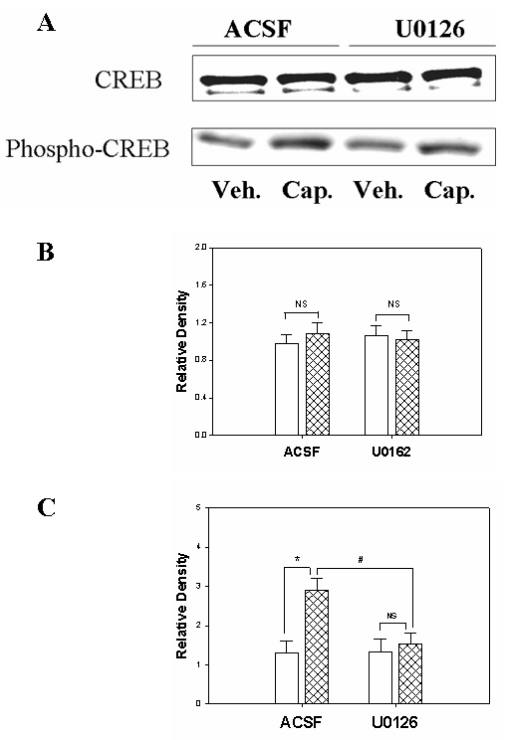
Detection of CREB protein and phosphorylated CREB in spinal cord in rats following vehicle or capsaicin injection and the effect of MAP kinase inhibitor, U0126. P*anel A*. Immunoblot data of CREB and phospho-CREB protein expression. P*anel B*. Bar graph summarizing the density of the immunoblot band of CREB protein. (NS, no significance between vehicle treatment vs. capsaicin treatment group in either ACSF- or U0126- treatment). P*anel C*. Bar graph demonstrating the density of the Western blot bands of phospho-CREB protein. (*, p < 0.05; the value from vehicle treatment vs. capsaicin treatment; #, p < 0.05, the value of ACSF-treated vs. U0126-treated animals treated with capsaicin; NS, no significance between vehicle vs. capsaicin treatment in groups with U0126 administration; n = 5 in each group). Open bar, vehicle group; hatched bar, capsaicin group.

**Figure 2:**
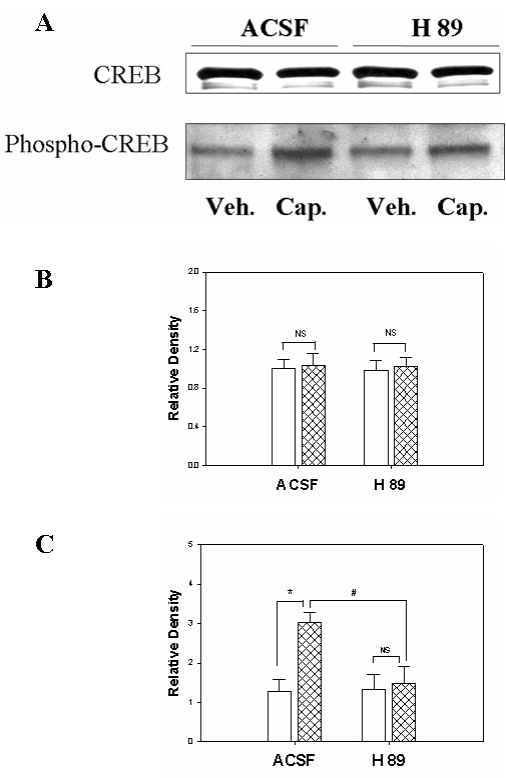
Expression of CREB protein and phospho-CREB protein in spinal cord in rats and the effect of intrathecal PKA inhibitor, H89, treatment. P*anel A*. Immunoblot bands of CREB and phospho-CREB protein expression. P*anel B*. Bar graph summarizing the density of the immunoblot CREB bands. (NS, no significance between vehicle treatment vs. capsaicin treatment group with either ACSF- or H89- treatment). P*anel C*. Bar graph showing the density of the immunoblotting bands of phospho-CREB protein. (*, p < 0.05; the value from vehicle treatment vs. capsaicin treatment; #, p < 0.05, the value of ACSF-treated vs. H89-treated animals after capsaicin injection; NS, no significance between vehicle vs. capsaicin treatment in groups with H89 intrathecal administration; n = 5 in each group). Open bar, vehicle group; hatched bar, capsaicin group.

**Figure 3:**
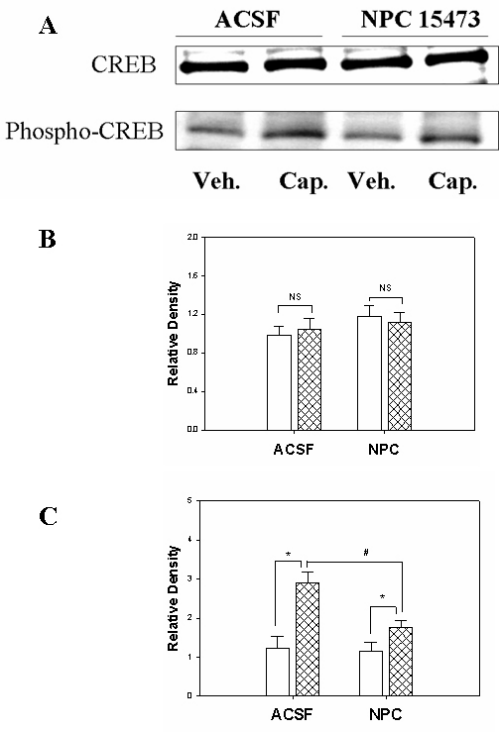
Expression of CREB and phosphorylated-CREB protein in spinal cord and the effect of intrathecal PKC inhibitor, NPC 15473, administration on their expression. P*anel A*. Western blot bands of CREB and phospho-CREB protein expression. P*anel B*. Bar graph showing the density of the immunoblot band of CREB protein. (NS, no significance between vehicle treatment vs. capsaicin treatment group with either ACSF- or NPC 15473- treatment). P*anel C*. Bar graph indicating the density of the immunoblotting band of phospho-CREB protein. (*, p < 0.05; the value from vehicle treatment vs. capsaicin treatment; #, p < 0.05, the value of ACSF-treated vs. NPC 15473-treated animals following capsaicin injection; n = 6 in each group). Open bar, vehicle group; hatched bar, capsaicin group.

Following capsaicin injection in four different treated groups of rats (ACSF-, U0126-, H89-, NPC 15473- treatment), the expression of CREB protein itself showed no significant changes in rats treated with ACSF when compared to the vehicle-treated groups (Figures 1B, 2B, 3B). Meanwhile, a significant enhancement of Ser-133 CREB phosphorylation (45KD) was found in the spinal cord tissue in the group of rats treated with ACSF. As we reported previously, the infusion of ACSF made no significant difference in the phospho-CREB expression when compared to that of naïve rats [6].

In our first experiment, we tested whether changes in CREB expression and CREB phosphorylation could be regulated by MAP kinase activity, The intrathecal application of U0126, a MEK 1/2 inhibitor (dissolved in 10% DMSO, 1 μg/ μl and diluted with PBS to a total volume of 10 μl), has been shown to reduce CREB phosphorylation in the spinal cord tissue, which is otherwise enhanced by capsaicin injection, when compared to the value from ACSF-treated animals (1.53 ± 0.29 vs. 2.91 ± 0.3, Figures 1A and 1C, p < 0.05). Within the group receiving U0126 for 30 minutes, the value of phospho-CREB expression in the capsaicin group demonstrated a higher level than that of the vehicle-injected group, which showed no significance (1.53 ± 0.29 vs. 1.34 ± 0.3, p > 0.05). However, ACSF treatment did not affect the increased phosphorylation of CREB protein in response to capsaicin injection (2.91 ± 0.3 vs. 1.31 ± 0.31, p < 0.05).

Next, we assessed the effect of protein kinase A (PKA) in mediating CREB phosphorylation since PKA is one of the important intracellular triggers for CREB activation. The intrathecal administration of the selective PKA inhibitor, H89 (10 μM for 30 minutes), was performed before inducing acute noxious stimulation with a capsaicin injection in rats. Results showed that changes in CREB protein expression were not found in either the ACSF-treated group or in H89-treated animals (Figure 2B). However, there was a significant increase in the phospho-CREB in ACSF-treated animals after capsaicin injection (1.29 ± 0.2 vs. 3.02 ± 0.25, p < 0.05). Moreover, this capsaicin-induced increase in CREB phosphorylation was inhibited by infusion of H89 when compared to that of ACSF-treated rats (1.49 ± 0.43 vs. 1.34 ± 0.37, p < 0.05, Figure 2C).

Finally, we demonstrated a role of protein kinase C (PKC) in regulating CREB phosphorylation in response to capsaicin injection. We infused the PKC inhibitor, NPC 15473, intrathecally before injection of capsaicin in rats. An enhancement of CREB phosphorylation was observed in capsaicin-treated animals when compared to the vehicle-treated group when ACSF was infused. However, the intrathecal infusion of NPC 15473 (20 mM for 30 minutes) significantly reduced the phosphorylation level of CREB following capsaicin injection. The decreased level showed a significant change when compared to that of rats receiving ACSF infusion (1.76 ± 0.2 vs. 2.89 ± 0.3, p < 0.05, Figure 3C). CREB protein itself did not respond to any treatment (Figure 3B).

## Discussion

The major findings in the current study demonstrated that intradermal capsaicin injection causes an increase in Ser-133 CREB phosphorylation in the dorsal horn and that the enhancement of CREB phosphorylation is regulated by the activity of MAP kinase, PKA and PKC.

A number of studies have shown that enhanced phosphorylation of CREB transcription factor could account for the potential contribution to long-term changes in the spinal processing of nociceptive information [6,13]. As one of important transcription factors, CREB is phosphorylated in response to stimuli-triggered calcium influx into post-synaptic neurons [14,21,22]. Our previous findings reported the role of nitric oxide system and CaM kinase II in the CREB regulation [13,25]. In this investigation, we report that noxious stimulation with capsaicin injection induces phosphorylation of CREB protein (Ser-133) in the spinal cord. This finding is similar to what we reported previously [6] and studies of other investigators using different stimulation regimens. In addition, we assessed the regulating mechanism by which intracellular protein kinase mediates CREB phosphorylation. The data demonstrated that the intrathecal treatment with inhibitors of MAP kinase, PKA or PKC blocked capsaicin-triggered CREB phosphorylation (at the Serine 133 site), confirming that not only c-AMP-dependent protein kinase A (PKA), but also ERK/MAP kinase and PKC, play roles in the mediation of CREB phosphorylation during the central processing of nociception.

Peripheral noxious stimulation, such as capsaicin injection, causes an increased responsiveness in spinal nociceptive neurons that involve the activation of glutamate receptors. This event produces a large influx of calcium into the nociceptive neurons, activating multiple intracellular protein kinase cascades, such as CaM kinase II, PKC, as well as MAP kinase [2,4,6,18,30-34]. The PKA, PKG and nitric oxide synthase systems were also found to be activated following increased cAMP and cGMP in nociceptive neurons during painful stimulation [2,22,35]. Enhanced phosphorylation of CREB through the activation of glutamate receptors and the above kinase cascades during central sensitization suggest a connection between CREB phosphorylation and the molecular mechanisms governing stimuli-induced CREB activation by protein kinase pathways. Investigators from other laboratories also noted that increased CREB phosphorylation was found in animals following different kinds of noxious stimulation [7,8,11,17].

Like other transcription factors, such as AP-1 proteins, c-*fos*, and c-*Jun*, phosphorylated CREB may participate in the downstream signal transduction cascade in the longer lasting changes in synaptic plasticity. CREB phosphorylation was reported in the transcription regulation of nociception-related genes, such as dynorphin, enkephalin and opioid receptors, during the activation of nociceptive neurons. A CREB binding site is found in the promoter regions of the dynorphin, enkephalin and opioid receptor genes following painful stimulation [14,16,20,28,35]. CREB phosphorylation is required for prolonged synaptic plasticity strengthening during central sensitization [2,21,24].

Recently, mitogen-activated protein (MAP)/extracellular signal-regulated kinase (ERK) kinase (MEK 1/2) was reported to play a critical role in spinal dorsal horn neurons in response to various painful stimuli [5,18,30-34]. Following noxious peripheral stimuli or c-Fiber volleys, an increased phosphorylation of ERK was observed in nociceptive neurons in rats and an inhibitor of ERK reduced the formalin-injection-induced nociceptive behavior [5,36]. Peripheral nerve injuries or noxious stimulation with various inflammatory agents, such as carrageenan, capsaicin, Complete Freund's Adjuvent (CFA), mustard oil and melittin (a major toxic peptide of whole bee venom) were reported to active the MAP/MEK kinase pathway [5,9,18,20,31,33,34][37]. Furthermore, the activation of MAP kinase pathways was related to the activation of several types of glutamate receptors (NMDA, AMPA and mGlu-R) in dorsal horn neurons, as well as other intracellular protein kinase cascades in the pathogenesis of nociceptive sensitization [9,18]. The activation of MAP kinase signaling was also reported to be involved in the transcriptional regulation of prodynorphin and neurokinin-1 (NK-1) gene products [5,14,16,35]. In our present study, we found that U0126, a MAP kinase inhibitor, significantly blocked CREB phosphorylation at Ser-133 in response to capsaicin injection. Another investigator reported a similar effect of U0126 in a different spinal cord slice preparation following C-fiber stimulation [9]. In an investigation involving in vitro preparation of cultured striatal neurons, the NMDA- and AMPA/kainate-induced CREB phosphorylation was blocked by MAP kinase inhibitor, U0126 [23]. These findings provide supportive evidence that the MAP kinase pathway is one of the important intracellular elements and may contribute to the nociceptive plasticity in central sensitization.

It is well recognized that both the PKA and the PKC pathways are involved in the central neurotransmission of nociception. In experimental animals with capsaicin injection induced-central sensitization, activation of the PKA or the PKC pathways by their activators enhanced the response of spinothalamic (STT) cells to mechanical stimuli [15,19] and inhibitors of PKA or PKC blocked the hypersensitivity of STT neurons. Experiments from behavioral studies also support the roles of PKA and PKC in the generation of spinal cord central sensitization [2,15,19]. Our study further demonstrates the roles of PKA and PKC in molecular mechanism of regulation of gene transcription in response to capsaicin injection, in which they mediate the phosphorylation of the transcription factor, CREB. However, as important regulators, both PKA and PKC actively contribute to the activity-dependent hippocampal synaptic plasticity by regulating CREB-dependent target genes. The present study supports the results from our earlier work on central sensitization, which is believed to be a spinal cord form of LTP [2].

In summary, the findings from our investigation provide strong evidence that MAP kinase, PKA and PKC participate in the regulation of CREB phosphorylation status in the spinal cord following peripheral noxious stimulation. These results also suggest a possible explanation for how the activated intracellular kinase cascades convey extracellular signals into the nucleus for subsequent transcription of plasticity-associated genes in the spinal cord.

## Methods

Male Sprague-Dawley rats (Harlan Sprague-Dawley, Houston, Texas) weighing 280–350 grams were used in this project. All experiments were performed in accordance with the ethical guidelines of the International Association for the Study of Pain and National Institutes of Health and were approved by the Institutional Animal Care and Use Committee of the University of Texas Medical Branch. Capsaicin (100 μg in 10 μl) was injected into the plantar surface of the left foot of rats following anesthesia with sodium pentobarbital (i.p., 50 mg/kg). For the intrathecal administration of agents, an intrathecal catheter (PE 32) was implanted in the spinal subarachnoid space. The other end was connected with PE10 and then PE20 tubing for a stepdown connection to a Hamilton syringe [4,6]. Briefly, a midline incision was made to expose the suboccipital region in the rats and the catheter was gently advanced ~7.4 cm into the spinal subarachnoid space. The tip of the catheter was placed at around the estimated level of the L4/5 spinal segments. The rats were returned to the facility for 5 days to allow them to recover from the surgery. On the day of the experiment, the catheter was connected to a 1 ml syringe, which was mounted on an infusion pump that kept a constant infusion rate of 1 μl/min for the delivery of inhibitors. U0126, a selective inhibitor of MEK 1/2 (1,4 -Diamino-2,3-dicyano-1,4-bis (2-aminophenylthio)-butadiene, Biosource, CA); H89, a protein kinase A inhibitor ((*N*-[2-((3-bromophenyl)-2-propenyl)amino)ethyl]-5-isoquinoline sulfonamide, HCL, Calbiochem., San Diego, CA); NPC15473, a protein kinase C inhibitor (2,6-diamino-*N*-([1-oxotridecyl)-2-piperidinyl]methyl)hexanamide, Alexis, San Diego, CA); and ACSF were infused. U0126 was dissolved in 10% DMSO (1 μg/μl) and diluted with PBS (total 10 μl). As we previously reported [25], the concentrations of NPC15473 and H89 were 20 mM and 10 μM, respectively. The specificity of the inhibitors was previously reported [5,19,25]. All of the agents were infused for 30 min. At 30-min post-infusion, the injection of capsaicin (100 μg in 10 μl) was performed. Spinal cord tissues were collected and Western blotting performed as previously reported [4,6,25]. Briefly, the animals were sacrificed at 30 min post-injection of capsaicin. The segments of the spinal cord tissues (L3–L6) were collected and immediately placed on a glass plate cooled with dry ice. The dorsal quadrants from the ipsilateral side were collected and immediately placed into liquid nitrogen. We were interested in detecting CREB phosphorylation in the ipsilateral side of the cord, since we previously reported that the biochemical changes in this side are more sensitive to capsaicin injection [4,6,26]. The tissue was homogenized with buffer containing 50 mol/l Tris buffer, pH 7.4(0.1 mol/l EGTA, 0.14 μl/ml β-mercapto-ethanol, 100 mol/l PMSF, and 0.2 mg/ml trypsin inhibitor). The protein concentration was measured by using a BCA kit (Pierce, Rockford, IL) and read on a microplate reader (Sun Bioscience).

Equal amounts of protein (60 μg) were loaded and size-fractionated by gel electrophoresis (SDS-PAGE) in a 4–20% Ready-Gel preparation and transferred onto a PVDF membrane (Bio-Rad, Hercules, CA). After incubating in blocking buffer, the membranes were incubated with primary polyclonal antibodies to CREB (1:800) or phospho-CREB (1:1000)at the Ser-133 site (Upstate Biotechnology, Lake Placid, NY) overnight at 4°C [17]. The anti-CREB is a polyclonal antibody from rabbits immunized with a synthetic peptide (C-SGAENQQSGDAAVTEAENQQ) corresponding to amino acids 5–24 of the human CREB. The antibody to phospho-CREB at the Ser-133 site was produced with another synthetic phospho-peptide corresponding to residues 123–136 (KRREILSRRPpSYRK). The blots were washed three times with PBS buffer and then incubated with HRP-conjugated anti-rabbit IgG (1: 5000; Santa Cruz, San Francisco, CA) in 5% (w/v) non-fat milk in PBS buffer. The membranes were enhanced with a chemiluminescence reagent (ECL kit, Amersham, Arlington Height, IL) and were exposed to the film. The expression of β-actin, as an internal control, was also measured with the same protocol in every group (monoclonal antibody against β-actin purchased from Sigma Co., St. Louis, MO). The density of the immunoreactive bands on the films was acquired by using a Doc-It Gel system and AlphaEase software. The densitometric units of bands of CREB or phospho-CREB were expressed relative to the values for β-actin. The significance of differences between groups treated with ACSF and protein inhibitors was calculated and compared with the Student's *t*-test [17]. P < 0.05 was considered significant. All data were expressed as mean ± S.E.M.
